# Netting barriers to prevent mosquito entry into houses in southern Mozambique: a pilot study

**DOI:** 10.1186/1475-2875-12-99

**Published:** 2013-03-16

**Authors:** Ayubo Kampango, Mauro Bragança, Bruno de Sousa, J Derek Charlwood

**Affiliations:** 1 MOZDAN (Mozambican-Danish Rural Malaria Project), PO Box 8, Morrumbene, Inhambane Province, Mozambique; 2 Centro de Malaria e Doenças Tropicais, Lisbon, Portugal; 3 Universidade Lusofona da Humanidade e Tecnologia, Campo Grande 376, Lisbon, Portugal; 4 Faculdade de Psicologia e Ciências da Educação, Universidade de Coimbra, Coimbra, Portugal; 5 DBL Centre for Health, Research and Development, University of Copenhagen, Fredriksberg, Denmark; 6 Present address: Laboratório de Entomologia, Instituto Nacional de Saúde (INS), Av. Eduardo Mondlane, No 1008, Maputo, Mozambique; 7 Present address: Liverpool School of Tropical Medicine, Pembroke Place, Liverpool, L3 5QA, UK

**Keywords:** Netting barriers, *Anopheles funestus*, *Anopheles gambiae s.l*, Abundance, House entry rate

## Abstract

**Background:**

One of the best ways to control the transmission of malaria is by breaking the vector-human link, either by reducing the effective population size of mosquitoes or avoiding infective bites. Reducing house entry rates in endophagic vectors by obstructing openings is one simple way of achieving this. Mosquito netting has previously been shown to have this effect. More recently different materials that could also be used have come onto the market. Therefore, a pilot study was conducted to investigate the protective effect of three types of material against *Anopheles funestus* and *Anopheles gambiae s.l* entry into village houses in Mozambique when applied over the large opening at the gables and both gables and eaves.

**Methods:**

A two-step intervention was implemented in which the gable ends of houses (the largest opening) were covered with one of three materials (four year old mosquito bed nets; locally purchased untreated shade cloth or deltamethrin-impregnated shade cloth) followed by covering both gable ends and eaves with material. Four experimental rounds (each of three weeks duration), from four houses randomly assigned to be a control or to receive one of the three intervention materials, were undertaken from March to August 2010 in the village of Furvela in southern Mozambique. Mosquito entry rates were assessed by light-trap collection and the efficacy of the different materials was determined in terms of incidence rate ratio (IRR), obtained through a Generalized Estimating Equations (GEE), of mosquito entry in a treated house compared to the untreated (control) house.

**Results:**

Altogether 9,692 *An. funestus* and 1,670 *An. gambiae s.l.* were collected. Houses treated with mosquito netting or the untreated shade cloth had 61.3% [IRR = 0.39 (0.32-0.46); P <0.0001] and 70% [IRR = 0.30 (0.25 – 0.37); P <0.001] fewer *An. funestus* in relation to untreated houses, but there was no difference in *An. funestus* in houses treated with the deltamethrin-impregnated shade cloth [IRR = 0.92 (0.76 –1.12); P = 0.4] compared to untreated houses. Houses treated with mosquito netting reduced entry rates of *An. gambiae s.l*, by 84% [IRR = 0.16 (0.10 – 0.25); P <0.001], whilst untreated shade cloth reduced entry rates by 69% [IRR = 0.31 (0.19 –0.53); P <0.001] and entry rates were reduced by 76% [IRR = 0.24 (0.15 0.38); P <0.001] in houses fitted with deltamethrin-impregnated shade cloth.

## Background

Most malaria transmission occurs inside houses when people are asleep. Improvements to houses were important in reducing malaria transmission in the early part of the last century [1,2]. Reducing mosquito entry rates into houses is a simple way of reducing transmission [3,4] and several methods to do this have been proposed, including building houses on stilts [5], using screens [6-9] or blocking the gap between the roof and walls [10,11].

In many rural areas of Africa, houses are simple structures without windows. Hence, mosquitoes, attracted to odour and carbon dioxide, tend to enter such houses via the gap between the roof and the walls [12,13]. This gap, in addition to allowing access to mosquitoes, provides illumination and ventilation and closing the opening with a solid barrier, as recommended by Kirby *et al.*[11], may not be acceptable in many cases. Airflow may also be increased through other openings if the larger openings are closed enabling mosquitoes to more easily find these secondary entry points.

An alternative system is to use semi-permeable barriers, such as mosquito netting, that allow air and light to enter the house but which prevent the mosquito from entering. Barriers have been shown to be effective in reducing malaria transmission in Burkina Faso [14] but, with the exception of work in The Gambia [7,9,11], despite the apparent advantages of such a technique, they have not been tested with much rigour elsewhere. Recently developed materials, such as UV-resistant polyethylene shade cloths for the protection of vegetables or vector control may be even more effective or better suited to cover house openings than mosquito netting. A pilot study was, therefore, conducted to determine if, when placed over the openings of houses in a village in southern Mozambique, different types of material of different permeability to airflow, affected mosquito entry rates as measured by light-trap collection. Since houses in this part of Mozambique have a large opening at the gable ends but smaller openings at their sides, the intervention was tested initially by closing only the large openings and subsequently closing all wall to roof openings.

## Methods

### Description of study site

The study was conducted between 24 March 2010 to 30 August 2010 in the village of Furvela (24°43’S, 35°18’E) in Morrumbene District, Inhambane Province, Mozambique. The village has been described by [15] and [16]. It lies circa 2 km from a mangrove-bordered coast to the west and is delineated by the Furvela river valley to the north. Both areas provide ample breeding sites for anopheline mosquitoes. A single rainy season occurs from October to March, when approximately 1,200 mm of rain falls, mostly in February and March. Daily mean temperatures vary between 18°C in July and 30°C in December. The majority of inhabitants live in rectangular houses built of reed with palm leaf roofs. Most houses do not have windows and have just a single door. There is usually an opening of 15 cm or more between the end gables and the roof. This provides ventilation, illumination and access for endophilic mosquitoes. The gap between the sides and the roof (the eaves) is smaller or non-existent. Malaria is endemic in the village. *Anopheles funestus* is the principal vector although *Anopheles gambiae, Anopheles arabiensis* and *Anopheles merus* also occur [15,16].

### The shade cloths

**Figure 1:**
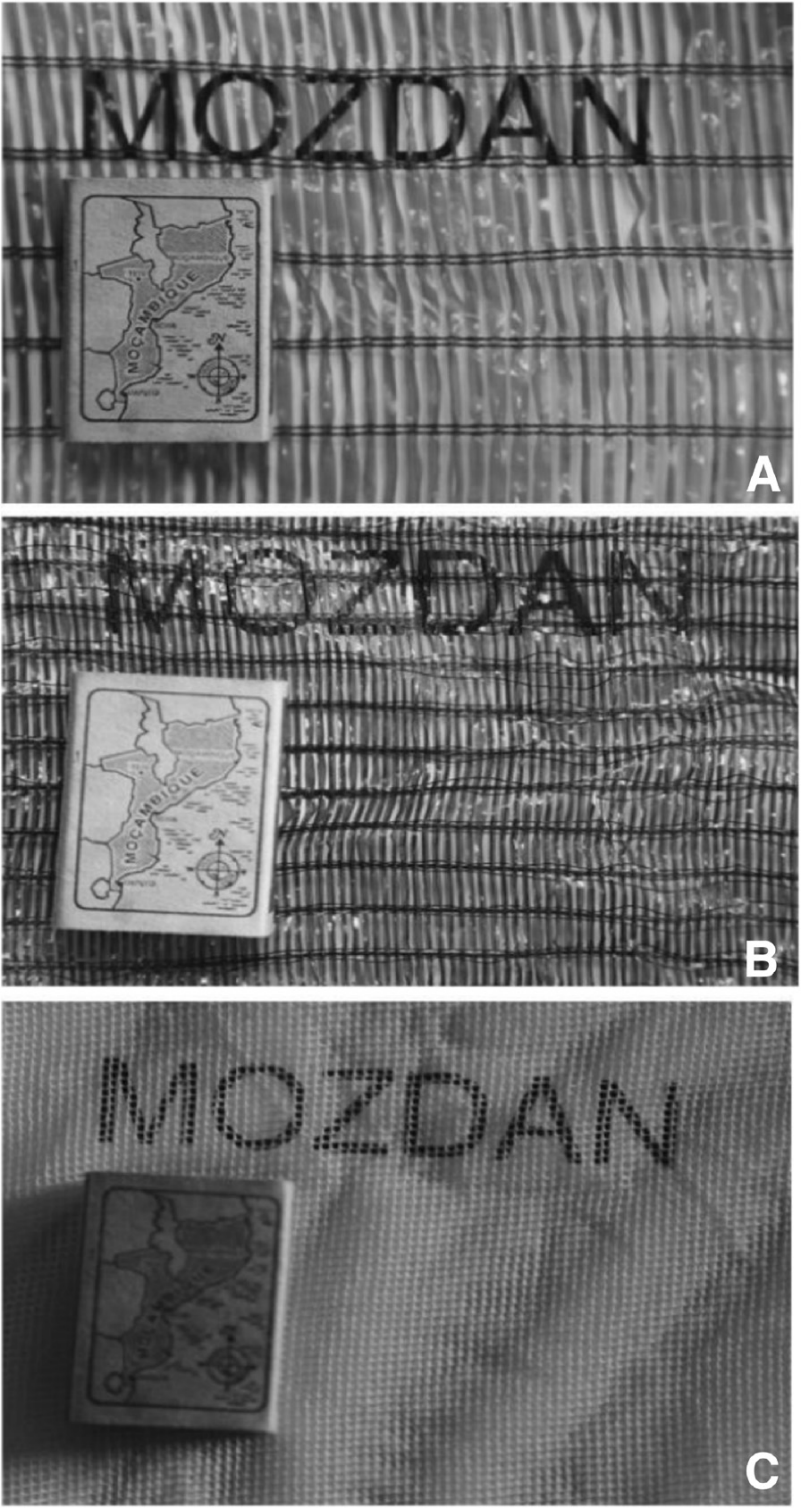
**A) Photograph of Zero Vector material used in the experiment. B**) Photograph of Maxixe material used in the experiment. **C**) Photograph of the netting used in the present experiment.

Three types of material that differed in their permeability to air were used in the experiment: 1) a deltamethrin-impregnated durable lining (henceforth Zero Vector cloth) manufactured by Vestergaard-Frandsen; 2) a locally purchased untreated-shade cloth used to protect growing vegetables (henceforth Maxixe cloth); and, 3) previously used Interceptor^®^ mosquito bed net (henceforth bed net). The Zero Vector cloth was made of fibres of polyethylene, weighing approximately 55 g/sq m. The cloth provides 50% shading and was impregnated with deltamethrin at 4.4 g/kg, incorporated into the yarn [17]. The gap size between fibres was circa 1.5 mm × 20 mm (Figure 1A). The weave of the Maxixe cloth, which was also made of polyethylene, was thicker than Zero Vector cloth and in addition to reducing airflow it reduced the amount of light coming into the house (Figure 1B). The Interceptor^®^ bed net was 75 denier, white polyester treated with the insecticide FENDOZIN^®^, a mixture of the insecticide alpha-cypermethrin with a binding polymer at a target dose of 6.7/kg or 200 mg/m^2^[18] (Figure 1C). The nets had been in use for four years prior to their application in the present experiment.

### Airflow measurement

A CDC light-trap, run from a freshly charged 6v 10 Ah sealed lead acid battery, attached to a 120 cm long, 15 × 15 cm cardboard tube was used to provide a constant airflow measured with a calibrated air velocity meter with a resolution of 0.01 m/s and an accuracy of ±3% (TSI TA440). The different fabrics were placed over the end of the opening of the cardboard tube and airflow reduction compared to the flow when no material was in place. For each fabric, airflow was recorded every five seconds over a five-minute period immediately in front and behind the cloth and mean velocities determined. Velocities were also measured between each replicate when no material was obstructing the opening of the funnel. The effect of the different materials on airflow was assessed as percentage reduction in flow. Air flow measurements were normally distributed (*P* > 0.05) therefore, two sample *t* tests were used to determine the significance of the difference between the mean air flow velocity before and after one the application of the material.

## Experimental design

### House selection criteria

In each of the four experimental rounds four rectangular houses with walls built of reed and roofs of palm leaves, which are the most common form of house in Furvela, were chosen for the experiment. Thus there were 16 houses used in the course of the work. Each experimental round of three weeks duration took place in a different part of the village. Houses used in the experiments were a minimum of 200 m from each other and at least two people slept in each house.

### Application of shade cloth inside houses

Three of the four houses in each experimental round were randomly assigned to receive one of the interventions: A- Zero Vector; B- Maxixe cloth, or, C- Bed net whilst the fourth served as the control. In the first week of each experimental round and prior to the application of the materials CDC light-traps were run for six consecutive days in each of the houses. In the second week the gable openings of the experimental houses were covered with the designated material (the level of intervention, henceforth designated gables ) [19]. Light-trap collections continued for a further six days, after which, in the third week of the experiment, the relevant material was also applied to the eaves of the treatment houses (the level of intervention henceforth designated gables + eaves). Mosquitoes were again collected for a further six days, giving a total of 18 days of sampling in each house for each experimental round.

### Statistical analysis

Statistical analysis was performed using the software IBM SPSS^®^ v. 20. [20]. Generalized Estimating Equations (GEE) with a negative binomial error distribution, , and a first-order autoregressive correlation structure were used to account, respectively for over dispersion of mosquito counts and serial correlation between repeated catches made in the same house over time [21,22]. Correlation between sequential observation in both *An. funestus* (r = 0.61) and *An. gambiae sl* (r = 0.54), suggests strong serial correlation. The variable ‘time’ was chosen as subjects and houses as within-subjects factor and GEEs were fitted separately to counts of the two vectors (*An. funestus* and *An*. *gambiae s.l.*) to determine the protective effect of three types of netting material against mosquito entry inside houses, when applied over gables and eaves openings. The efficacy of the material was determined in terms of incidence rate ratio (IRR) of mosquito entry in treated houses compared to the control house [23]. In all the analyses the control house (in which no intervention was in place) was the reference group and a 5% level was used to determine statistical significance.

### Ethical considerations

The study took place under the aegis of the joint INS-DBL project ‘Turning houses into traps for mosquitoes’ which obtained ethical clearance from the National Bioethics Committee of Mozambique on the 2^nd^ April 2001 (Ref: 056/CNBS/01). Householders were informed about the purpose of the experiment and were told that if they did not like the intervention it would later be removed. Only once consent had been obtained were houses surveyed, the sizes of openings determined and collections inaugurated.

## Results

### Airflow data

Zero Vector cloth had least effect on airflow. This was followed by the mosquito netting, the mean reduction in flow being 16% and 26% of the control respectively. The Maxixe cloth, on the other hand, reduced airflow by 87% (see Table 1).

**Table 1:** **Difference of air flow velocity (m.s**^**-1**^**) through a tube before and after one of its ends be blocked by shade cloths**

	**Measurements**	**Mean air flow (±se)**		
**Material**	**(N)**	**Without netting**	**With netting**	**P values**
Bed net	94	0.91 (±0.02)	0.65 (±0.003)	<0.001
Maxixe	94	0.92 (±0.004)	0.048 (±0.002)	<0.001
Zero vector	94	0.86 (±0.006)	0.70 (±0.005)	<0.001

### Mosquito composition and abundance

A total of 13,639 mosquitoes were caught during the experiment. *Anopheles funestus* was by far the most abundant species, comprising 71.1% (9,692) of all mosquitoes collected, followed by *An. gambiae* s.l. (henceforth called *An. gambiae*) which comprised 12.2% of the total. Three hundred and fifty seven (82%) of the 437 *An. gambiae* s.l. identified to species from 2002 to 2004 were *An. gambiae*, 71 were *An. arabiensis* and nine were *An. merus* (João Pinto, personal communication). Hence, the majority of the *An. gambiae* s.l. were *An. gambiae* s.s. but there was a significant proportion of *An. arabiensis* in the village. Samples of *An. funestus* identified to species from 2010 suggested that *An. funestus* s.s is the only member of the group in the village (James Austin *unpub data*). Other mosquitoes collected included *Culex spp* and *Anopheles tenebrosus* (5.4% and 4.2% of the total, respectively). *Uranotaenia palmeirimi* (2.8%) and *Mansonia africana* (2.3%) were the other species collected in any number.

**Figure 2:**
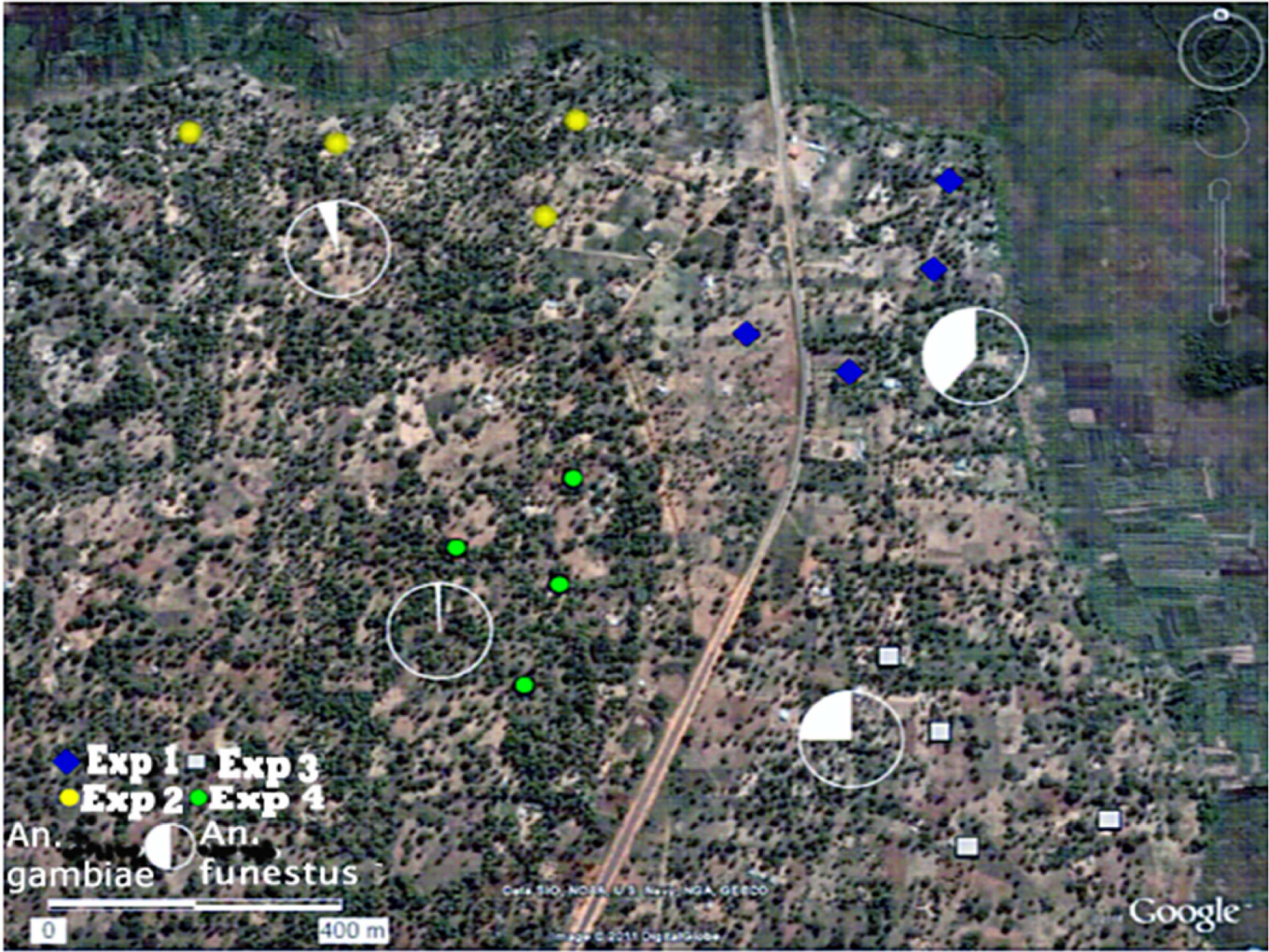
**Google map showing the location of the experimented houses in Furvela village. **The pie charts show the relative proportion of *Anopheles funestus *and *Anopheles gambiae *collected in each area. Symbols: Exp 1, Exp 2, Exp 3 and Exp 4 denote experimental rounds 1,2,3 and 4, respectively.

A considerable variation in the density of mosquitoes collected was observed, both in time and place. Significantly more *An. funestus* were collected in the final experimental round from the southern area of the village compared to the other three areas (*P* <0.001), that is: northern, north-eastern and south-eastern areas (Figure 2, Table 1). At the same time this area, had fewer *An. gambiae* (P <0.001) than the other areas (Figure 2, Table 2).

**Table 2:** **Total and mean marginal number (Estimated by GEE model) of *Anopheles funestus *collected by level of intervention and type of shade cloth; and the efficacy, expressed in terms of incidence rate ratio (IRR) of mosquito entry in treated houses compared to untreated houses, of the materials against the entry of *Anopheles funestus *into houses in Furvela village**

**Factors**	**Number collected**	**Mean (95% Wald CI)**	**IRR (95% Wald CI)**	***P *value**
No intervention	3874	28.9 (24.9 – 33.5)	1	
Gables	2873	21.9 (19.4 – 24.9)	0.75 (0.62 – 0,91)	0.004
Gable and Eaves	2901	23.4 (19.8 – 27.6)	0.8 (0.64 – 1.01)	0.056
No shadecloth	3372	43.4 (38.0 – 49.6)	1	
Zero vector	3476	39.6 (34.0 – 46.0)	0.92 (0.76 – 1.12)	0.40
Maxixe cloth	1220	13.0 (10.7 – 15.7)	0.3 (0.25 – 0.37)	<0.001
Bed net	1580	16.3 (14.4 – 18.4)	0.39 (0.32 – 0.46)	<0.001

### Efficacy of materials on mosquito entry

There were three collection days in which the malfunctioning of light-trap in the previous night was noticed by the house occupants and reported to a member of the team. Collections from those days were considered as being missing data and were not included in the analysis giving a total of 285 suitable samples instead of the anticipated 288 sampling occasions.

#### Anopheles funestus

Table 2 presents the results of the efficacy of three materials on *An. funestus* entry in treated houses in relation to Control houses. Results suggest that, entry rates of *An. funestus* were significantly reduced when the material was fitted over the gables of houses and that extending the intervention over eaves did not improve the protective effect. Results indicate that after the intervention was in place there were 70% [IRR = 0.30 (0.25-0.37); P <0.001] and 61.3% [IRR = 0.39 (0.32-0.46); P <0.001] fewer *An. funestus* entering houses treated with Maxixe cloth and mosquito bed net, respectively compared to untreated (control) houses. The number of mosquitoes entering houses treated with Zero Vector was not significantly different to those entering untreated houses [IRR = 0.92 (0.76-1.12); P = 0.4].

#### Anopheles gambiae

All of the materials significantly reduced entry of *An. gambiae* (*P* <0.001) (Table 3). There were 84% fewer *An. gambiae* [IRR = 0.16 (0.10–0.25); P <0.001] inside houses treated with mosquito netting, 76% [IRR = 0.24 (0.15 0.38); P <0.001] in houses fitted with Zero Vector and 69% (IRR = 0.31 (0.19–0.53); P <0.001) in houses treated with Maxixe cloth (Table 3). None of the sixteen households wanted the material removed from their houses at the end of the study.

**Table 3:** **Total and mean marginal number (Estimated by GEE model) of *Anopheles gambiae *s.l. collected by level of intervention and type of shade cloth and, the efficacy, expressed in terms of incidence rate ratio (IRR) of mosquito entry in treated houses compared to untreated houses, of the material against the entry of *Anopheles gambiae *into houses in Furvela village**

**Factors**	**Number collected**	**Mean (95% Wald CI)**	**IRR (95% Wald CI)**	***P *value**
No intervention	1064	6.52 (4.88 – 8.72)	1	
Gables	386	1.64 (1.23 – 2.18)	0.17 (0.11 – 0.27)	<0.001
Gable and Eaves	220	1.12 (0.77 – 1.63)	0.25 (0.17 – 0.37)	<0.001
No shadecloth	595	6.88 (4.98 – 9.51)	1	
Zero vector	397	1.66 (1.18 – 2.34)	0.24 (0.15 – 0.38)	<0.001
Maxixe cloth	458	2.13 (1.48 – 3.08)	0.31 (0.19 – 0.50)	<0.001
Bed net	220	1.12 (0.75 – 1.67)	0.16 (0.10 – 0.25)	<0.001

## Discussion

The results clearly indicate that application of the used mosquito nets or the Maxixe shade cloth significantly reduced the total number of *An. funestus* entering houses and that all of the materials used in the study reduced the numbers of *An. gambiae* entering. Houses do, however, have to be relatively well built in the first place and the door must fit securely since gaps around badly fitting doors can easily become entry points for hungry female mosquitoes even when the gables and eaves are covered. In the present experiment the effect of covering only the gable ends or the complete house differed between vectors. Covering the whole house improved the effect against *An. gambiae* but made no difference to the effect against *An. funestus*. There are two possible explanations for the enhanced effect against *An. gambiae*: either the two mosquitoes differ in the way that they enter houses, more *An. gambiae* going through openings in addition to the gables, or the effect had something to do with the insecticide on two of the three materials (Zero Vector and Bed net). The bed nets had been in use for four years and had been washed a number of times so it is not known how much insecticide remained on the material. Since the *An. funestus* in Furvela are resistant to the pyrethroids used on these materials and resist knockdown for more than 30 minutes when exposed to fresh active ingredient (Charlwood and Kampango, unpublished data) the difference is likely to be due to differing house entry strategies between the two vectors. Unfortunately, the resistance status of the *An. gambiae* is not known. The efficacy of the one material not impregnated with insecticide also implies that observed reductions were due to physical rather than chemical effects.

Notwithstanding the fact that this was only a pilot study, and for scientific exactitude a much larger randomized control trial would be useful, the results are sufficiently encouraging to suggest to villagers that covering the openings to their houses with netting or shade cloth is a good thing to do. As with bed nets the real problem (whether the material has insecticide or not) is how to get the intervention in widespread use. Although the intervention by itself may not significantly reduce transmission in a village such as Furvela, where inoculation rates are among the highest recorded (Charlwood*,* unpublished), it is an intervention well worth advocating. It is available, cheap and low-tech and, once in place, does not require further input from the householder. The use of old mosquito nets for this purpose would seem apposite since they are the most readily available material throughout much of the tropics and can be installed with a few nails or wire on a ‘do it yourself’ basis. Their use would also solve the problem of what to do with old, no longer used, nets.

## Competing interests

The authors declare that they have no competing interests.

## Authors’ contributions

AK did the fieldwork, analysed the data and helped write the paper. MF and BdS helped design the study, helped analyse the data, and helped write the paper. JDC designed the study and wrote the paper. All authors read and approved the final manuscript.
